# Is it the subcoracoid impingement or the subacromial impingement that tears the subscapularis tendon? A comparison of the MRI findings of the operated and healthy shoulders of the patients

**DOI:** 10.55730/1300-0144.5582

**Published:** 2022-10-22

**Authors:** Mehmet ÇETİNKAYA, Ahmet Yiğit KAPTAN, Coşkun ULUCAKÖY, Özlem ORHAN, Murat TOPAL, Tacettin AYANOĞLU, Ulunay KANATLI

**Affiliations:** 1 Department of Orthopaedics & Traumatology, Başakşehir Çam ve Sakura City Hospital, İstanbul, Turkey; 2 Department of Orthopaedics & Traumatology, Faculty of Medicine, Harran University, Şanlıurfa, Turkey; 3 Department of Orthopaedics & Traumatology, Dr. Abdurrahman Yurtaslan Onkoloji Training and Research Hospital, Ankara, Turkey; 4 Department of Orthopaedics & Traumatology, Faculty of Medicine, Kastamonu University, Kastamonu, Turkey; 5 Department of Orthopaedics & Traumatology, Faculty of Medicine, Abant İzzet Baysal University, Bolu, Turkey; 6 Department of Orthopaedics & Traumatology, Faculty of Medicine, Gazi University, Ankara, Turkey

**Keywords:** Subscapularis tendon, subcoracoid impingement, subacromial impingement, subscapularis tear

## Abstract

**Background/aim:**

The purpose of this study is to investigate whether the etiological factors accepted as causes of idiopathic subscapularis tears are true or not when the comparison is made with the opposite side healthy shoulder of the patients who underwent arthroscopic repair for an isolated subscapularis tear.

**Materials and methods:**

Sixteen patients who underwent shoulder arthroscopy between February 2016 and January 2018 and were diagnosed with isolated subscapularis tear were evaluated. The coracohumeral distance (CHDax), coracoid overlap (CO), and tuberculum minus cysts (TMC) were evaluated on the axial images of the MRI studies while the acromiohumeral distance (AHDsag), CHDsag, and subscapularis tendon slip number (STSN) on the sagittal oblique images and the AHDcor and SLAP lesion on the coronal oblique images. Degeneration of the coracoacromial ligament was evaluated during arthroscopy.

**Results:**

The mean CHDsag (11.26–10.08), CHDax (10.63–9.98), CO (14.2–15.43), AHDsag (8–7.66), and AHDcor (7.65–7.68) measurements (operated side-healthy side, respectively) were statistically similar (p > 0.05). No statistically significant difference was found between TMC and STSN in healthy and operated shoulders (p > 0.05). There was mild coracoacromial ligament fraying in 4 (25%) and obvious coracoacromial ligament fraying in 8 (50%) which indicated subacromial impingement in 75% of the patients.

**Conclusion:**

The parameters of the coracoid process did not reveal any significant difference between the operated (for an isolated subscapularis tear) and opposite-side healthy shoulders of the patients. However, coracoacromial ligament degeneration was present in 75% of the patients.

## 1. Introduction

Rotator cuff tears, one of the most common pathologies of the shoulder, may occur due to intrinsic and extrinsic factors or trauma. The degenerative tears of the rotator cuff tendons are most commonly seen in the supraspinatus and infraspinatus tendons, while the subacromial impingement (SAI) syndrome is often the etiologic factor [1]. The tears of the subscapularis tendon are rare and often accompany other rotator cuff tears. Studies on etiological factors have also increased with the understanding of the importance of this tendon in recent years. In the etiology of subscapularis tendon rupture, one common area for etiology includes subcoracoid impingement syndrome. Subcoracoid impingement (SCI) can be classified as traumatic, iatrogenic, and idiopathic [2]. The etiology of the former two is apparent. However, the etiology of idiopathic SCI is still under debate.

There are several studies in the literature assessing the anatomic parameters of the coracoid process and proximal humerus by computed tomography (CT), magnetic resonance imaging (MRI), and cadaveric investigations [3–8]. Although these studies have evaluated the morphology, 3-dimensional structure, and subcoracoid interval measurements of the coracoid projection, the presence of idiopathic subcoracoid syndrome are still controversial. For this reason, the questions about the cause of tears of subscapularis tendon tears due to nontraumatic reasons have not been answered yet.

The purpose of this study is to investigate whether the etiological factors accepted as causes of idiopathic subscapularis tears are actual or not when the comparison is made with the opposite side healthy shoulder of the patients who underwent arthroscopic repair for an isolated subscapularis tear. This paper attempted to add further to the present data on idiopathic SCI and aimed to enlighten the issue of whether the existence of this syndrome is actual or not, thus focusing on the idiopathic and isolated cases and the healthy opposite side shoulder of the patients. The hypothesis of this study is that subcoracoid impingement is related with coracoid parameters other than subacromial parameters when compared with a healthy opposite shoulder.

## 2. Materials and methods

This cross-sectional study investigates the differences between the healthy and unhealthy shoulders of the patients who underwent shoulder arthroscopy for an isolated degenerative subscapularis tear. The Institutional Review Board approved the data accumulation and the study. All patients signed the consent forms to be included in the study. In accordance with the ethical principles of the Declaration of Helsinki; the *Power Analysis* determined the study group size to include a minimum number of patients because the radiologic (MRI) assessment of the healthy shoulder was required for this investigation.

### 2.1. Patients

The power analysis test revealed the number of patients needed to reject the null hypothesis that the failure rates are equal with probability (power) β = 0.8 (1-Type 2 error) and α = 0.05 (Type 1 error) as 16 when it was assumed that the mean CHD was 10.4 mm in healthy shoulders and 8.6 mm in operated shoulders with a standard deviation of ±1.8 according to a previous study [9].

The data of the patients were started to be collected in February 2016, and the 16th patient was operated on in January 2018. All of the procedures were performed in the same institute by the senior surgeon of the study with a 15-year of experience in shoulder arthroscopy.

Patients were excluded if there is a history of prior surgery, those with symptoms started acutely right after a trauma in the vicinity of the involved shoulder (including fractures, dislocations, and falling), osteoarthritis, inflammatory joint disease, hemophilic arthritis, crystalloid deposition diseases, and those with one of these disorders in the opposite side healthy shoulder. Until reaching the mapped outpatient number, which was 16, one patient with a traumatic subscapularis tear and two patients who refused to undergo a second MRI for their opposite side healthy shoulders were not included in the study. Moreover, patients with any accompanying pathologic finding revealed during the arthroscopy were excluded, except for the SLAP lesion and biceps tendinitis, which were stated previously to accompany almost always the subscapularis tears [10,11]. The diagnosis of the subscapularis tear was based on a physical examination followed by radiographs and MRI. The Belly-press, Belly-off, Lift-off, Lift-off lag, and Bear-hug tests were the examination tests intended to assess the subscapularis tendon integrity, and the Neer and Hawkins tests were for the subacromial impingement [12–14]. The MRI findings suspicious for subscapularis tear were an interruption of the tendon continuity, signal hyperintensities such as fluid or near-fluid signal on T2 axial images, and fatty infiltration on the T1 sagittal oblique images. Tendons with a clear continuity and a hypointense in the substance were assumed healthy.

### 2.2. Arthroscopic procedure

**Figure 1:**
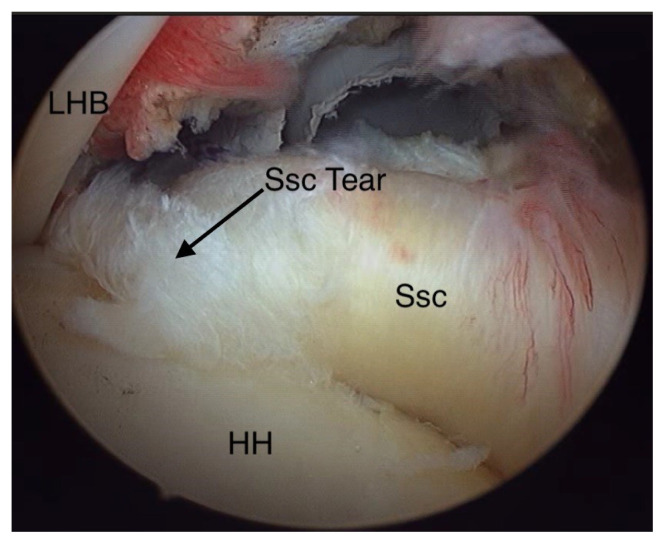
Subscapularis tendon tear.

The position of the patients was semilateral decubitus, in which the patients were allowed to rotate 20–30° posteriorly to place the glenoid fossa parallel to the floor. The anesthesia option was a single inter-scalene block or general anesthesia with or without an inter-scalene block. Following the sterilization with iodine solution and draping of the shoulder, the arm was positioned in a 45° abduction and 15° forward flexion under 10 lb longitudinal traction. The standard posterior portal was used for the initial shoulder examination through a 30° rigid arthroscope. Additional portals were constituted according to the planned interventions decided after diagnosing the intra-articular pathologies with an initial arthroscopic examination through the posterior portal. A spinal needle was used for the examination of the subscapularis initially before constituting any additional portal. If any intra-articular pathology was diagnosed, the second portal was constituted through the rotator interval or laterally through the full-thickness superior rotator cuff tear. An arthroscopic examination probe was used for a further examination of the intra-articular structures and the subscapularis tendon as well. If the subscapularis tear repair was decided, the anterolateral portal which is preadjusted with a spinal needle is placed through the superior-lateral corner of the subscapularis tendon coursing medially parallel to it. A tendon that has a folding or an undulation near its insertion area and which can be lifted off of the tuberculum minus humeri was assumed to be a subscapularis tear (Figure 1) and was classified according to the Lafosse classification system [15]. The arthroscopic classification of the SAI syndrome was made according to the Royal Berkshire Hospital’s classification described by Levy et al. [16]. Grade 0 was normal coracoacromial ligament appearance, Grade 1 was minor fraying, Grade 2 was major fraying, and Grade 3 was the visualization of the bare bone under the coracoacromial ligament.

### 2.3. MRI procedure and the measurements on MRI

MRI examinations were performed with a dedicated shoulder coil on a 1.5 T system (Signa, HiSpeed, General Electric Medical Systems, Milwaukee, Wisconsin) in the supine position of patients as the arm was lying beside the body in neutral rotation. The imaging protocol included oblique coronal T1-weighted (TR/TE: 600/16) and fat-suppressed intermediate (T2-weighted) (TR/TE: 3000/56), oblique coronal T1-weighted [TR/TE: 500/16] and fat-suppressed intermediate (TR/TE: 3000/56), and T2-weighted axial (TR/TE: 500/15, flip angle: 30) images. The field of view was 18 cm, the matrix was 192–384 × 256, and the slice thickness/inter-slice gap was 2 mm in all sequences.

**Figure 2:**
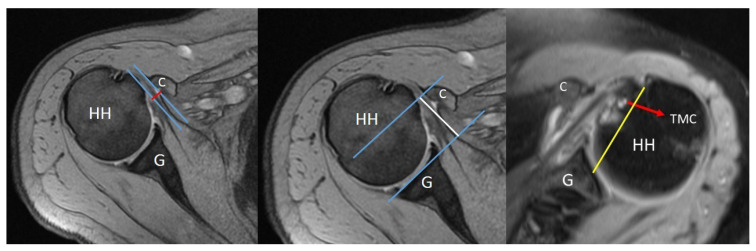
Coracohumeral distance (CHDax-red line), coracoid overlap (CO-white line) measurement, and tuberculum minus cysts (TMC-red arrow) on axial sections of magnetic resonance imaging scans.

**Figure 3:**
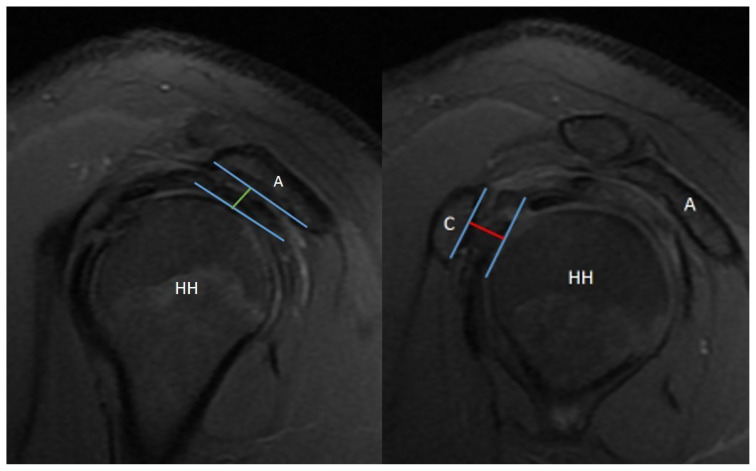
Acromiohumeral distance (AHDsag-green line) and coracohumeral distance (CHDsag-red line) on sagittal oblique sections of magnetic resonance imaging scans.

**Figure 4:**
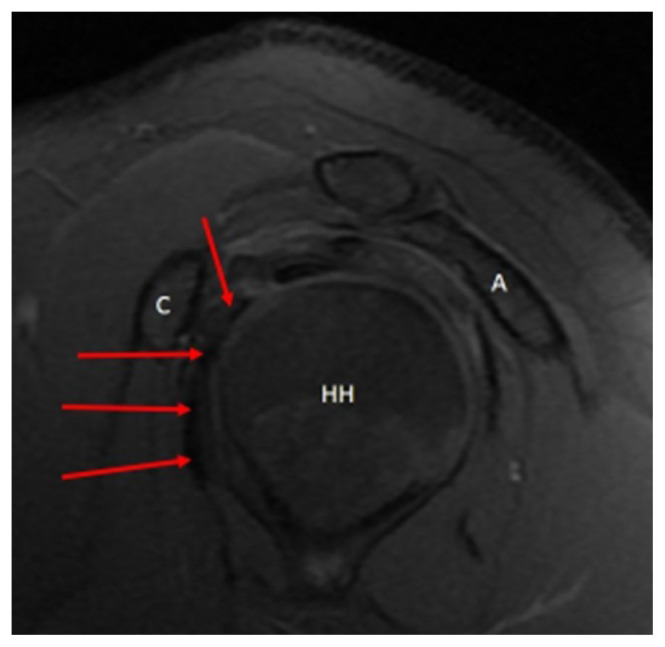
Subscapularis tendon slip number (STSN-red arrows) on sagittal oblique sections of magnetic resonance imaging scans.

**Figure 5:**
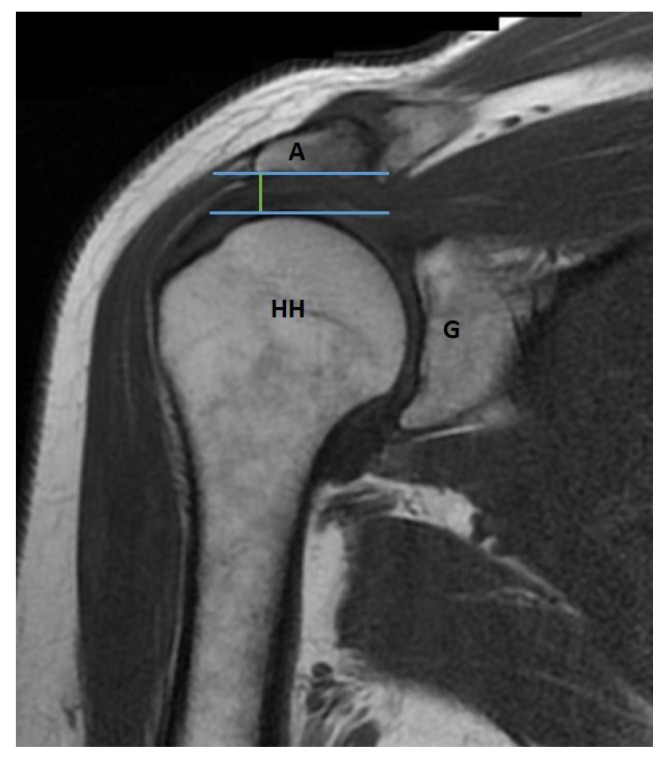
Acromiohumeral distance (AHDsag-green line) on sagittal oblique sections of magnetic resonance imaging scans.

All of the measurements were performed by an orthopedic surgeon who is dedicated to shoulder arthroscopy. The inter-observer and intra-observer correlations were not calculated in this study since they are already reported previously by several studies. The coracohumeral distance (CHDax), coracoid overlap (CO), and tuberculum minus cysts (TMC) were evaluated on the axial images of the MRI studies (Figure 2) while the acromiohumeral distance (AHDsag), CHDsag, (Figure 3) and subscapularis tendon slip number (STSN) (Figure 4) on the sagittal oblique images and the AHDcor (Figure 5) and SLAP lesion on the coronal oblique images. The CHD is the shortest distance between the outer cortices of the humeral head and the coracoid process on transverse and/or sagittal oblique sections of MRI [17]. The CO is the shortest distance on the axial images between the line parallel to the glenoid fossa and the most prominent aspect of the coracoid process [17]. The TMC existence was approved if there was any cyst formation over 2 mm diameter anterior to the line between the center of the biceps groove and the center of the glenoid fossa on the axial images of MRI (Figure 2) [11]. The AHD is the shortest distance between the inferior aspect of the acromion and the superior aspect of the humeral head on coronal and sagittal oblique scans of the MRI [18]. The STSN has been previously evaluated with the naked eye in cadavers and with MRI in patients [19–21]. The subscapularis tendon origins from the subscapularis fossa of the scapula as 1–6 strips. These strips tend to merge as they course laterally. Since the STSN was previously shown to be a potential contributing factor for an SCI, the strip number was assessed at the coracohumeral space on axial MRI images [6]. The STSN number found on MRI scans was grouped according to those with three or fewer strips and those with four or more strips [6]. The diagnosis of a SLAP lesion on MRI was made on coronal oblique images. Any synovial fluid under the bicipital-labral complex at the superior aspect of the glenoid or a synovial cyst placed medial to the superior glenoid pole are assumed to be the signs of a SLAP lesion. The measurements and STSN evaluation were made on T1-weighted scans, while the SLAP, TMC, and subscapularis integrity evaluations were on T2-weighted scans.

### 2.4. Statistical analysis

The continuous variables, including CHD, AHD, and CO, were analyzed with Wilcoxon’s Test, and the categorical variables, including TMC, STSN, and SLAP lesion, were analyzed with the Crosstabs and Pearson’s χ2 *test*. For all comparisons, statistical significance was set at the value of p < 0.05 level (2-tailed). Statistical analyses were performed with the SPSS (Statistical Package for Social Sciences) software (Version 21.0; SPSS Inc, Chicago, IL).

## 3. Results

There were ten female and six male patients in the study. The mean age was 49.8 ± 8.7 (min-max: 24–61). The operated side was left (all nondominant side) in 10 patients and right (all dominant side) in 6 patients. Nine patients had positive Neer signs and four had both positive Neer and Hawkins signs. According to the Lafosse classification, there were 10 Type 2 and 6 Type 3 tears. A SLAP 2 lesion was present in 11 (68%) patients. Only 5 of those had evidence of SLAP lesion on MRI, and six patients had false-negative MRI findings. There was no arthroscopic SAI finding in 4 of 16 patients, while there was mild fraying in 4 (25%) and noticeable fraying in 8 (50%) which indicated SAI in 75% of the patients.

The mean CHDsag, CHDax, CO, AHDsag, and AHDcor measurements (operated side-healthy side, respectively) were statistically similar when compared to the Wilcoxon test (p > 0.05) (Table). A TMC was found in two patients, and there was no TMC on the healthy shoulders of these two, while a TMC was found in 5 patients in healthy shoulders but none in the operated shoulders of these 5 (p > 0.05). The STSN was evaluated by both Crosstabs (groups), and no significant difference was found between the operated and healthy shoulders (p > 0.05).

## 4. Discussion

The SCI syndrome has been investigated a number of times in a number of studies with a number of parameters. There have been reported results opposite to one another, even in papers with quite similar study designs [2, 6, 7, 9, 17, 22, 23]. The instinct was mostly towards the existence of this syndrome which paves the way for subscapularis tendon tear. There are studies conducted with a high number of patients investigating several parameters related to the coracoid process and its relationship with the humeral head and glenoid [6,7], and there are also reports comparing the patients with subscapularis tears and those without in terms of SCI [6,24]. The most important aspect of the current study was its design which focused on comparing the operated shoulders of patients to the opposite side of healthy shoulders to find why that shoulder affected the other. However, after a pretty much workload, there has been found no significant difference in any of the parameters to be an etiologic factor of the isolated subscapularis tears, except the SAI which was found 75% in this cohort.

The subcoracoid distance narrowing may occur with the anterior-superior translation of the humeral head [25]. The supraspinatus tear results in superior migration of the humeral head, and the coracoid process extends along inferiorly and laterally towards the tuberculum minus humeri after originating from the scapula and extending superiorly; thus, a potential superior humeral excursion may put the subscapularis tendon at risk by decreasing the distance between the coracoid process and the humeral head [6]. However, the decrease in coracohumeral distance does not mean the SCI syndrome is present all the time. According to a study, there are patients with a CHD under even 6 mm but with no subscapularis disorder [6]. As a result, all of these comments and data take us to the question of “Does the SCI really exist? If it does, then why there are studies in the literature stating no further improvement following the coracoplasty for SCI” [26]. On the other hand, the improved outcomes reported by several studies should not be imprudently accepted as strong evidence of the coracoplasty effect since the coracoplasty procedures were performed in addition to subscapularis repair, which prevents to interpret the improved outcomes whether they are by means of coracoplasty or tendon repair [2, 25, 27–29].

The major symptom of the SCI is anterior shoulder pain which aggravates by forward-flexion and internal rotation of the shoulder and radiates to the arm as in the SAI by Hawkins sign [7]. Yamamoto et al. published their study revealing the humeral impingement area with the coracohumeral ligament was supraspinatus with Neer sign and subscapularis with the Hawkins sign, which indicates a potential risk for subscapularis tear caused by subacromial impingement [13]. This finding was consistent with the current study reporting coracoacromial ligament degeneration as 75%.

It is still not clear yet whether the source of pain is the rotator cuff tendon tear or the secondary changes, because we know there are asymptomatic individuals with a full-thickness supraspinatus tear, particularly those with intact rotator cable as Burkhart et al. described [30]. Conversely, even with a partial tear or smallest-sized full-thickness defects, a supraspinatus tear may cause severe pain, especially if the SAI syndrome develops [31]. The relationship of the subscapularis tendon tear with the biceps tendon disorders may be similar to that of the supraspinatus tendon tear and SAI because the biceps tendon pathologies, which is the pain generator of the shoulder, such as anterior dislocation and tendinitis/degeneration are frequently encountered copathologies with subscapularis tendon tears [11,32,33].

The strong relationship of the cysts with the rotator cuff tendons, as well as the TMCs with the subscapularis tendon, was previously reported by several studies [22,23,34]. In the current study, the TMC was fewer in the operated shoulder than in the healthy ones. Subscapularis tears are frequently accompanied by superior rotator cuff tears which were previously reported as 83% by two different studies [11,35]. Therefore, the cysts may be occurring mostly when the subscapularis tear is together with the superior rotator cuff tear. Another odd may be the younger-aged population of the current study (49.8) because we know the cysts are more frequent in the elderly [34].

The SLAP lesion rate was 68%. It was found rather rare when compared to the previous study which reported 91% SLAP 2 lesions with subscapularis tears [11]. It was probably due to the younger-aged patients and only the isolated cases in the current study because it was reported that rotator cuff tears are strongly associated with SLAP 2 lesions [36–38]. Therefore, adding the supraspinatus and infraspinatus tears to the subscapularis may be an increasing factor in the rate of SLAP lesions.

The STSN was previously reported to be mostly three or fewer in subscapularis tears by Cetinkaya et al.’s study [6], in which the authors commented that the fewer number of tendon strips warrant more tendon volume per strip, which may be a reason for a coracohumeral impingement. They also stated that the force distribution to a higher number of tendons may reduce the force applied per strip which may be a preventive phenomenon for subscapularis tendons. The current study revealed no difference in terms of strip number, which was concordant with the expected because the comparison was with the other shoulder of the same individual.

In the literature, there are ongoing discussions about CO, CHD, and AHD measurements and whether these are related to subcoracoid impingement. The measurements of those parameters have been made with various methods. Gerber et al. measured CHDax in 47 healthy shoulders with CT and reported that the mean CHDax was 8.7 mm for the arm in adduction and 6.8 mm in flexion and internal rotation [39]. Friedman et al. used MRI to measure CHDax in normal volunteers and patients with shoulder pain [40]. They reported CHDax was 11 mm in healthy volunteers and 5.5 mm in patients with the maximum internal rotation. Richards et al. measured CHDax with MRI in patients with arthroscopically proved subscapularis tears and in patients without any rotator cuff, subscapularis, or subcoracoid pathology [24]. They reported that CHDax was 5.0 ± 1.7 mm in the subscapularis group and 10.0 ± 1.3 mm in the control group. Conflicting with most literature studies, there are also studies which reported that CHDax was not a predictive value for subcoracoid impingement [6]. In the current study, the mean CHDsag was 11.26 and 10.08 mm, while the CHDax was 10.63 and 9.98 mm for operated and healthy shoulders of the patients, respectively, which was not significantly different. Giaroli et al. also measured CHDsag and CHDax in the subscapularis and nonsubscapularis groups [41]. They reported CHDsag as 11.8 and 10.7 and CHDax as 9.9 and 8.6 for nonsubscapularis and subscapularis tear groups, respectively. They concluded that the CHD measurement has a limited diagnostic role in subcoracoid measurement.

CO is the amount of lateral extension of the coracoid projection according to the glenoid plane. CHD may decrease with internal rotation of the shoulder, and excess CO may lead to narrowing [4,39,40]. Hatta et al. made 3D models of the coracoid process and proximal humerus using MRI of four healthy volunteer shoulders in four-arm positions including an internal rotation at 0°, 45°, and 90° of flexion, and 90° of flexion with maximum horizontal adduction [8]. They reported that the lateral part of the coracoid was the closest portion of the coracoid process to the proximal humerus in all shoulder positions. A previous study reported that CO and STSN were related more significantly to subcoracoid impingement than CHD and that the CO was 24.01 ± 4.9 mm and 21.29 ± 4.58 mm for the study and control groups, respectively [6]. However, considering the size difference between patients, CO may differ between patients. Therefore, in another study, the ratio of the humeral head diameter and CO was calculated to avoid the change in the size of the coracoid process in relation to the size of the patient [42]. However, there was no significant difference between arthroscopically proved isolated subscapularis tear and isolated supraspinatus tear groups. Also, in the current study, CO was not significantly different between the healthy and operated shoulders of the patients.

### 4.1. Limitations

The number of patients was very low in order to limit the number of unnecessary diagnostic procedures. The coracoacromial ligament degeneration classification was present only for the operated side shoulders. It was impossible to provide that data from healthy shoulders. The measurements made on MRI are controversial since any change in the patient position during the MRI study may change the results, despite the strict positioning rules. In this study, only the isolated cases were included. Therefore, subscapularis tears accompanied by supraspinatus tears secondary to the SCI are excluded, which may constitute a bias in patient sampling.

The parameters of the coracoid process did not reveal any significant difference between the operated (for an isolated subscapularis tear) and opposite-side healthy shoulders of the patients. However, coracoacromial ligament degeneration was present in 75% of the patients.

## Figures and Tables

**Table:** The mean values of the measurements on magnetic resonance imaging scans. CHDsag: Coracohumeral distance on sagittal oblique images, CHDax: Coracohumeral distance on axial images, AHDsag: Acromiohumeral distance on sagittal oblique images, AHDcor: Acromiohumeral distance on coronal oblique images, CO: Coracoid overlap, SD: Standard deviation.

	Operated shoulder (± SD)	Healthy shoulder (± SD)	p-value
**CHDsag**	11.26 ± 3.52	10.08 ± 4.38	>0.05
**CHDax**	10.68 ± 3.79	9.98 ± 3.02	>0.05
**AHDsag**	8 ± 1.69	7.66 ± 1.2	>0.05
**AHDcor**	7.65 ± 1.65	7.68 ± 1.2	>0.05
**CO**	14.2 ± 4.6	15.43 ± 4.52	>0.05
